# Opium in Irritable and Anemic States of the Brain in Fever

**Published:** 1853-10

**Authors:** Humphry Sandwith

**Affiliations:** Physician to the Hull General Infirmary, and Lecturer on the Practice of Medicine in the Hull School of Medicine


*Opium in Irritable and Anæmic States of the Brain in Fever.* By Humphry Sandwith, M.D., Physician to the Hull General Infirmary, and Lecturer on the Practice of Medicine in the Hull School of Medicine.—"The Employment of opiates in cerebral affections," says Dr. Holland, "is a question of much interest and various difficulty;" and "there is a great scope for farther research on this subject, as on all that relates to disorders of the brain, and a strong presumption that opium is capable here of larger and more beneficial application than has yet been given to it." His subsequent remarks, in the same article, "On the Use of Opiates," embrace, but are not restricted to, its use in fever, and may be consulted with advantage. Meanwhile, the profession owe Dr. Latham a large debt of gratitude for his masterly sketch of those irritable and anæmic states of the brain in fever, which demand the cautious use of this powerful narcotic. His brief but comprehensive paper on the subject, published twenty years ago, is still a beacon to guide us in a path, which his observant genius first irradiated.

The class of cases of purely irritable states of the brain is to be discrminated, as Dr. Latham shows less by any *series* of symptoms flowing from the brain, than from the *single* symptom of a state of protracted wakefulness. Nor is the wakefulness pathognomonic *per se*, but to warrant the use of opium, it must occur in combination with an irritable state of the nervous system, induced either by depressing moral agencies, or by the physically exhausting one of alcohol. The fever may be mild, and "exhibit a *sort of contrast* with the existing affection of the brain;" or it may correspond in severity with the sensorial disturbance up to a certain point, and then the symptoms referable to the brain outrun the febrile phenomena. In the latter case, though vascular over-action may have been kept in check by general or local bleeding, still the sensorial disturbance progresses. "As other symptoms are relieved, the delirium is even aggravated." We are thus presented with two forms of irritable brain in fever, —the one being marked by simple wakefulness with no other cerebral symptom, and the other by wakefulness coupled with symptoms of high sensorial excitement. Both varieties are "incident only to those, whose habits and mode of living have been calculated to do an abiding injury to the nervous system, and who have been long actually suffering from such injury."

The same acute observer, however, recognizes another variety of sensorial disturbance in fever, which is obviously associated with anæmia We shall quote his words.

"Again, I have seen the sensorial affections incident to fever, which require opium for their cure, manifest themselves in another form. There has been high vascular action from the first; and *large depletion* has been required to subdue it and to guard particular organs, and especially the brain, from injury. Under such treatment, all has gone on successfully, and the patient has reached the point of convalescence, with a soft pulse, a cleaning tongue, no pain, and refreshing sleep for two or three days; when suddenly (the tongue, the pulse, and all other circumstances continuing the same) some strangeness of manner has arisen, and then the wildest delirium, and then the unrestrained passage of the evacuations. I have known the transition from such a state of *convalescence* to such a state of peril, take place in a few hours; and I have known the patient again brought back to a state of convalescence in twenty-four hours by a moderate dose of opium. This is a rare form of disease, but one in which, when it does occur, opium is eminently indicated."

Now here was anæmic condition of the brain, but not to so frightful an extent as in the case to which I beg leave now to call the reader's attention; and I may add, that its very extent suggested, not "a moderate dose of opium," but the liberal use of the remedy.

CASE.—Mrs. T., a rather delicate woman, about thirty years of age, fell into fever during the second week of November, 1848, when near the close of the third month of pregnancy. Her abode was in the vicinity of open and offensive ditches; and it was soon evident that the attack would prove severe. The case ran on nearly three weeks, with symptoms of grave and increasing disturbance of the sensorium, and other indications of low typhoid fever. By careful management, indeed, had she not been pregnant, the disease might probably have evinced no symptoms of more than usual danger. On the 2d day of December, the irritation of the great nervous centres palpably interfered with gestation, and abortion was the speedy consequence. The process unhappily was accompanied with a very profuse hemorrhage, which ceased indeed with the expulsion of the fetus and placenta, but which in twelve hours had brought the patient into imminent peril. Along with continued sensorial disturbance, there were the signs of incipient collapse, as manifested by a rapid and fluttering pulse, unequally diffused animal heat and laborious respiration. Moderate stimulation by wine had been latterly allowed; but it now became necessary both to give ammonia, and augment the dose of alcohol. The extreme restlessness, subsultus, and other alarming symptoms demanded, however, a cordial, on which more reliance could be placed than even on the stimulus of brandy. Life, in short, was now in extreme peril. Calling to mind the marvellous examples of the virtues of opium in uterine hemorrhage *without fever*, as recorded by Dr. Stuart in the *Medico-Chirurgical Transactions*, as well as its power to sustain life in the fearful struggles of angina pectoris, and reasoning from the analogy of its virtues *in fever*, when the circulation is at the same time depressed by antimony, as also in delirium tremens, when excessive and habitual intoxication may be presumed to have exhausted the vital energies of the brain, I determined on an attempt to steady the heart, restore the capillary circulation, and calm the irritation of the nervous centres by a full dose of opium. Mr. Millin, the surgeon with whom I was attending in consultation, fully concurred with me in these views. We accordingly gave a draught containing a hundred drops of laudanum. The effect justified our most sanguine hopes. Sleep ensued, the circulation rallied; every symptom of cerebral irritation subsided, fever broke up, and convalescence was speedily established.

The present short practical paper contemplates chiefly that condition of the brain in fever, in which an anæmic state of its vessels warrants us in availing ourselves of the stimulant properties of opium, with a view to maintain the equilibrium of the cerebral circulation. In such cases the use of the drug is salutary, chiefly as it favors an amount of congestion essential to healthy sleep. Not that its effects on vascular structure are its only merits; for it is fair to argue with Dr. Holland, that as " narcotic substances have effects, locally applied, on nervous sensibility," so also " there can be little doubt, that in sleep it is the same singular influence, extended more widely over this part of organizaton, and reaching through the cerebral parts of it, the higher faculties of our being." But in anæmic conditions of the brain in fever, the use of opium seems to be salutary, we repeat, chiefly as it favors an amount of congestion essential to healthy sleep. " It is certain, that the states of sleep and coma frequently graduate into each other, in such way as to show that the proximate physical conditions are nearly the same in both;" for " one degree of pressure seems essential to perfect and uniform sleep, while a greater degree, without other alteration of state, assumes more or less the character of disease." While such changes in the circulation of the head are obviously concerned in influenciug the functions of consciousness and voli'ion, it is equally manifest that an anæmic state of the vessels is precisely the one calculated to disarm the narcotic of its dangerous properties. But for that anæmic state, the cerebral capillaries might be goaded on by a full dose of opium into fatal coma. The necessity of a healthy amount of congestion and consequent pressure, however essential to the production of sleep, may be inferred also from considerations regarding the nutrition of the organ. " The sleep of animals," Dr. Carpenter tells us, " consists not in a state of diminshed energy of the *nutritive* functions, but in the cessation of the *sensorial* activity, dependent upon a suspension of the functional power of certain parts of the nervous system, during which there is reason to believe that the nutritive and reparative operations of those organs go on with even augmented rapidity." The value of sleep in this view, and the importance of a healthy circulation of the materials for nutrition, considering the wasting effects of sensorial hyper-activity in fever, are self-evident.

On witnessing such striking displays of the remedial virtues of opium, as in the case related above, one is fain to break out with Sydenham "in praise of the great God, the giver of all good things, who hath granted to the human race, as a comfort in their afflictions, no medicine of the value of opium, either in regard to the number of diseases that it can control, or its efficacy in extirpating them!" After all, most of its value depends on the discrimination with which it is prescribed. When injudiciously administered, as in sthenic cerebral excitement or in the improper arrest of diarrhœa in certain states of fever, it has been observed to produce phrenitis, epilepsy, and coma; so also, when indiscriminately prescribed in delirium tremens, its employment has occasionally been followed by apoplexy. Great and marvelous, therefore, as are the virtues of opium in a variety of diseases, and admirable as are its soothing qualities in several of the forms of cerebral disorder in fever itself, yet let no man venture to prescribe it for the latter (whether in large or small doses) in the dark or at random. It is a sharp-edged tool, and of such fearful potency, that, if it fulfils not a curative intention, it will probably destroy life. There is no instance, in the whole range of practical medicine, more imperatively demanding a sure diagnosis. In short, our warrant to prescribe it hinges on our ability to ascertain precisely that condition of the brain which alone will admit of its safe employment.

It was a rare sagacity which led Dr. Graves to employ tartar-emetic in combination with opium in those cases both of idiopathic fever and delirium tremens, in which the narcotic would probably stimulate to over-congestion of the brain, but for the depressing action of the mineral on the heart and capillary circulation. This complex practice finds its parallel in the analogous operation of opium in states of anæmia in fever.

Medical science is more advantaged, perhaps, by defining the circumstances to which our known remedies are applicable, than by exploring the resources of nature for others. Not only is great discrimination necessary in deciding on the cases which demand the use of opium, but also in regulating the doses adapted to each variety. Little can be added to Dr. Latham's admirable directions. Our rule of conduct indeed is suggested by the degree of sensorial excitement. "Simple wakefulness may be gently lulled to sleep by a few drops of laudanum, but wild delirium requires to be mastered and (as it were) forced into repose by a much larger dose." Opium, however, goes much farther; and therefore, a much less dose is required in quelling asthenic sensorial disorder, combined with fever, than when it exists alone. Five minims in the milder cases, and twenty in the graver, may be considered minimum and maximum doses in fever. Much, after all, must be left to our vigilance in watching its effects, and to our discretion in judging when to desist, and when and in what doses to repeat the remedy. There are yet other "cases where the iudications for the employment of opium are *doubtful*." We shall describe the variety of sensorial disturbance in fever in Dr. Latham's own words:—

"Wild delirium, and long wakefulness, and a circulation weak and fluttering, seem to call for a considerable dose of opium. Yet, withal, there is a *certain jerk* in the pulse, so that we cannot help suspecting that the bloodvessels have something to do with the sensorial excitement. Under such circumstances, I have certainly seen twenty minims of laudanum produce tranquil sleep, from which the patient has awoke quite a new man; but I have also seen the same quantity produce a fatal coma, from which he has never been roused."

Dr. Latham's recommendation, therefore, is in so dubious a case, and to avoid "striking a heavy blow in the dark," to administer a small dose at intervals of one hour or two, "so as to stop short of actual mischief at the first glimpse of its approach, or be led by a plain earnest of benefit to push the remedy to its full and consummate effect." Judicious as is this advice, we cannot but think, that on this mode of treatment of cases, in which, on Dr. Latham's own showing, "the indications for the employment of opium are doubtful," any practice which aims at once to keep down vascular action and soothe the nervous system is a real improvement. And such, we need scarcely add, is the complex method of Dr. Graves.

It only remains that we should revert to the *anæmic* type of sensorial disturbance in fever, in that milder variety of it which, as described by Dr. Latham, we placed in contrast with a much graver form, " a moderate dose of opium," he tells us, suffices to change a state of peril to one of convalescence. But his remarks do not contemplate so frightful a form of anæmia in fever as that of which we have given an example, and which might result equally from a large intestinal, nasal, or bronchial hemorrhage, as from uterine. He speaks of heroic doses of opium only " in extreme cases of delirium tremens," while twenty minims of the tincture, he asserts, are quite sufficient for the purpose of subduing " the very same symptoms, carried to the greatest extremity, *when combined with fever*." But while " a moderate dose" suffices to relieve the less urgent anæmic forms of the disease, the case related above as having occured to my own observation, seems to sanction a bolder practice in the perilous cases of an extremely ex-sanguine condition of the brain in fever, coupled with the exhaustion resulting from protracted sensorial excitement.—*Association Med. Jour.*

